# Comparative Network Analysis of Preterm vs. Full-Term Infant-Mother Interactions

**DOI:** 10.1371/journal.pone.0067183

**Published:** 2013-06-21

**Authors:** Lilla Sipos, Benedicte Mengel Pers, Magda Kalmár, Ildikó Tóth, Sandeep Krishna, Mogens H. Jensen, Szabolcs Semsey

**Affiliations:** 1 Institute of Psychology, Eötvös Loránd University, Budapest, Hungary; 2 CMOL, Niels Bohr Institute, University of Copenhagen, Copenhagen, Denmark; 3 Institute of Cognitive Neuroscience and Psychology, Hungarian Academy of Sciences, Budapest, Hungary; 4 National Centre for Biological Sciences, Bangalore, India; Cinvestav-Merida, Mexico

## Abstract

Several studies have reported that interactions of mothers with preterm infants show differential characteristics compared to that of mothers with full-term infants. Interaction of preterm dyads is often reported as less harmonious. However, observations and explanations concerning the underlying mechanisms are inconsistent. In this work 30 preterm and 42 full-term mother-infant dyads were observed at one year of age. Free play interactions were videotaped and coded using a micro-analytic coding system. The video records were coded at one second resolution and studied by a novel approach using network analysis tools. The advantage of our approach is that it reveals the patterns of behavioral transitions in the interactions. We found that the most frequent behavioral transitions are the same in the two groups. However, we have identified several high and lower frequency transitions which occur significantly more often in the preterm or full-term group. Our analysis also suggests that the variability of behavioral transitions is significantly higher in the preterm group. This higher variability is mostly resulted from the diversity of transitions involving non-harmonious behaviors. We have identified a maladaptive pattern in the maternal behavior in the preterm group, involving intrusiveness and disengagement. Application of the approach reported in this paper to longitudinal data could elucidate whether these maladaptive maternal behavioral changes place the infant at risk for later emotional, cognitive and behavioral disturbance.

## Introduction

Understanding and predicting human behavior has been a central question in the history of mankind. Recently, interest turned to quantitative analysis of human activities using mathematical models and network tools, addressing temporal and structural features of human communication [1], [2]. To gain new insights into one of the most fundamental parts of human activities, we compare preterm and full-term babies’ and mothers’ behaviors in dyadic situations. Prematurity is not an illness and does not unconditionally cause a developmental delay; however, preterm babies are at risk of impaired cognitive and social development [3]–[5]. A preterm infant’s developmental prospects depend on risk- and protective factors. Understanding and predicting the long-term outcome of development have been addressed by applying perinatal risk scales [6] and by analyzing environmental factors such as socio-economic status and the quality of life [7]. Because the explanatory power of these approaches was found to be weak, research focus turned toward caregiver-infant interactions which have been found to contribute to the developmental outcome through complex transactions between infant characteristics and caregiver behaviors [8]–[10]. A growing amount of evidence suggests that maternal behaviors toward preterm babies may have differential characteristics, which are either adaptive or maladaptive in light of the preterm baby’s atypical needs [11].

The premature baby’s developmental lag and the weaker self-regulation requires a higher degree of adaptation from the mother [12].

Failure of adaptation to the baby’s atypical needs can put the interaction at risk. Neonatal neurological functions normally developing in intrauterine conditions have to develop outside. This overburdens the under-developed nervous system of the very young baby. Preterm infants are often difficult to interact with: they tend to be less organized, less optimally alert, less responsive to stimulation and provide less clear signals [13] which makes the interaction less pleasant or rewarding for the dyads. For instance, Crnic et al [14] found that mothers of preterm infants smile less often and their infants show less positive affect throughout the first year than full-terms do. The differences were most noticeable at 12 months.

Premature birth may find the parents unprepared for welcoming the baby both in physical and psychological terms. The maternal attitudes are influenced by a host of negative emotions, like disappointment, feeling of guilt, resentment, or anxiety about the baby’s survival and potential impairment as well as by the often shocking appearance of the preterm baby, the long separation, and the behavioral manifestations of the immature, stressed nervous system [13]. However, the reported data on the characteristics of the preterm mothers’ behaviors are inconsistent. In some studies the mothers of preterm infants were more active and responsive than mothers of full-term infants [14], [15], whereas other authors found the opposite: the preterm mothers were less active, less sensitive and responsive, and expressed fewer emotions [10], [13], [16].

Various reasons may account for the apparent inconsistency, e.g. the degree of immaturity and perinatal complications in the infant, maternal preparedness and support, the infant’s age at the observation, and the context of interaction [17], [18].

In addition, there are distinct ways of how data are derived from the observed events. The majority of studies on mother-infant interactions used global rating scales [19], [20], which may be helpful in detecting certain features of the interaction but do not catch patterns in the sequences of behaviors. Microanalytic (frame by frame) coding systems, in contrast, are suitable for recording bidirectional transactions [21], [22]. These systems preserve the chronology of events, and also allow observation of rare events. Microanalytic coding systems have been developed for the analysis of different interactions, including physician-patient, couples, and mother infant interactions [23]–[25].

**Figure 1:**
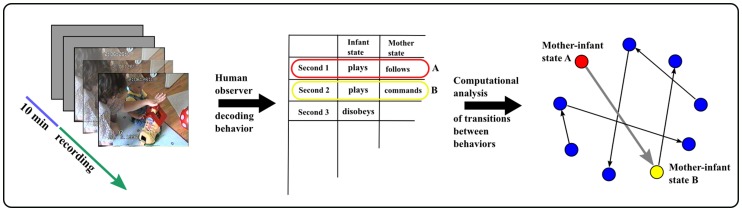
Design of the experimental approach. Preterm and full-term infants’ and mothers’ interactions were videotaped in dyadic situations and coded micro-analytically. Coded data were analyzed through formation of complex interaction networks and by identification of transition patterns between combined infant/mother states. The participants shown on the photograph have given written informed consent, as outlined in the PLOS consent form, to publication of their photograph.

In this paper we present a comparative study on the early mother-infant relationship. Our novel approach is summarized in Figure 1. This approach involves utilization of network analysis tools, which have recently been applied for quantitative analysis of human activities, e.g. addressing temporal and structural features of human communication [1], [2]. Preterm and full-term infants’ and mothers’ behaviors were observed in dyadic situations and coded micro-analytically. Coded data were analyzed through forming interaction networks and identifying transition patterns between combined infant/mother states in order to capture the key characteristics of preterm and full-term infant-mother interactions. Our network approach reveals the interaction pattern of all behavioral states and can also highlight potential interaction paths. In-depth analysis of a vast observational material by our novel approach provides new insights into human interactions which could not be found by the conventional analysis tools used in psychology.

## Methods

### Design

The data presented and analyzed in this paper are a subset (age of 1 year ±2 weeks) of the data from a prospective longitudinal quasi experiment aiming at detecting the determinants of developmental outcome of preterm children. In this study the preterm group is compared to a control group containing full-term mother-infant dyads. The study is a quasi experiment because it lacks random assignment of subjects to groups [26]. The research protocol was approved by the Research Ethics Committee of the Institute of Psychology of the Hungarian Academy of Sciences. Signed informed consents were obtained from the parents for participating in the study, as well as from the parents on behalf of their children that they also participated in the study.

### Subjects

Seventy-two singleton infants and their mothers participated in the study. Thirty of these infants were born preterm, at 28–33 weeks of gestation (mean GA 30.9 weeks, *SD* 1.5 weeks), with birth weights of 800–1990 grams (mean BW 1437 grams, *SD* 260 grams). The children possessed no congenital abnormalities or obvious sensory deficiencies, and their perinatal course was free of severe complications. The ages of the preterm infants were corrected according to their expected birthday. Risk scores on the Parmelee Obstetric and Postnatal Complication Scales [27] ranged between 6–17 (mean 10.4, SD 2.9), and they were regarded by the neonatologists as low- to moderate risk babies. The male/female ratio was 50/50 (none of the perinatal variables were related to gender). Mothers of preterm babies were recruited soon after the childbirth in a Neonatal Intensive Care Unit in Budapest (Hungary).

The gestational age range for the preterm infants was chosen with certain considerations in mind. After 28 weeks of gestation, with good perinatal care and if the organism is otherwise healthy, the degree of maturation enables the central nervous system to adapt the vital autonomic processes to the extrauterine conditions without life-threatening difficulties. On the other hand, it is an extremely important period in the development of alertness and state regulation, and in this respect these preterms are expected to be still markedly different from the full-term neonates.

The comparison group of 42 healthy full-term infants (GA >37 weeks, mean BW 3421 g, SD 374.3 g, range 2650–4350 g, 52% boys, 48% girls) and their mothers were selected from the subjects of the Budapest Parent-Infant Study [28]. The mean age of the mothers was 28.3 years in the preterm group (range: 20–42), and 26.6 years in the comparison group (range: 19–34). The two groups were comparable in demographic variables (living conditions, fathers’ education, parents’ profession). However, mothers of full-term babies had somewhat higher levels of education χ^2^(3, N = 72) = 14.39, p<0.05.

### Procedure

Mother-infant dyads were observed at the infant’s age of 12 months in a play situation. To reduce potential reactivity [29], observations were made at the subjects’ home. Observational sessions were recorded by a female researcher using a handheld video camera. Each visit began with a familiarization period, lasting about 10 minutes. Subsequently the mother was asked to play with her infant as she ordinarily would, and to disregard the researcher’s presence as much as possible. Mean length of interactions was 415 second (∼7 min) (*SD* 118 sec).

### Behavioral Recordings

The videotaped events were coded separately for mother and infant resulting in two parallel behavioral state streams. Using a mutually exclusive and exhaustive micro-analytic category system, every second of the behavior was coded with a single category within each mother/infant behavioral stream. Hence within each mother/infant behavioral stream the beginning of a new behavioral state necessarily implies the end of the previous behavioral state.

### Behavioral Categories

Interactions were discerned in aspects of (1) whether there is a joint activity or not, (2) how a harmonious/disharmonious play interaction is developed and broken up, (3) how infant and mother are related to each other: (i) whose play idea is accepted or who leads the interaction, (ii) how leadership gets accepted or refused by the other. The categories were the following:

Infant: 1: *plays* (plays with a toy of his/her interest); 2: *explores* (searches for/approaches new toy); 3: *obeys* (the child passively acts in accordance to the mother’s commands, verbal or non-verbal initiative, interference or physical control, without showing either negative or positive emotional reaction); 4: *cooperates* (the infant follows maternal verbal or physical interactive actions, initiations with an interested and/or positive emotional expression (e.g. smile, laugh, positive vocalization, gesture of excitement); 5: *defies* (actively opposes the mother’s idea/command); 6: *neglects* (ignores mother or her ideas, does not comply with the mother’s command but does not oppose explicitly); 7: *passive* (is not involved in any activity); 8: *other* (none of the above categories).

Mother: 10: *other* (none of the categories below); 11: *follows* (follows the infant’s playing activity, she adapts herself to the infant, they focus on the same thing, mother is involved); 12: *enriches* (enriches the infant’s play with her own ideas, but does not change toy/game, elaborates the infant’s play, shows a new aspect of how to use a toy); 13: *physically forces* (physically forces or prevents the infant from doing something); 14: *commands* (verbally demands the infant to do something); 15: *directs attention* (intrusively directs the infant’s attention. She insists on her own idea, irrespective of the infant’s involvement in doing something else); 16: *interrupts* (interrupts the infant’s play activity with anything else other than directing the infant’s attention to another toy, e.g. cleans the nose, adjusts clothes of the infant, etc.); 17: *passive* (not doing anything and being uninvolved); 18: *neglects* (not playing with the infant, and actively doing something else); 19: *inappropriate* (any behavior not satisfying the infant’s obvious need, expressing disappointment about the infant’s behavior, or expressing developmentally unreachable expectation towards the infant); 20: *manipulates toy* (not playing but manipulating the toy to promote the infant’s activity, e.g. assembling a toy).

Based on previous reports [30], [31] we considered the interaction to be the most harmonious when infant was engaged in a play (1) and the mother followed or enriched his activity (11, 12). More generally, interaction was considered to be smooth if the mother (11, 12) or the infant (3, 4) adjusted to the partner’s idea. When leadership was not accepted by the other, conflict occurred and interaction was found disharmonious (infant: 5, 6, mother: 13, 14, 15). Neglecting behavior (6, 18) expressed lack of joint activity and ignorance toward the other person.

Inter-rater reliability was established by coding 14% of the sample by two independent coders. Time-unit kappa κ = 0.82 was based on whether the coders agreed with the behavior category within 2 seconds, and computed by GSEQ [32].

### Construction of Interaction Networks

In order to get an insight into the dyadic nature of the interaction, i.e. how the behavior of one party affects the actions and reactions of the other party, we applied network analysis tools. A behavioral transition was defined as a change in either the infant’s and/or the mother’s behavioral state. These transitions were extracted from the coded behavioral streams using custom MatLab (MathWorks, version R2010b) scripts.

**Figure 2:**
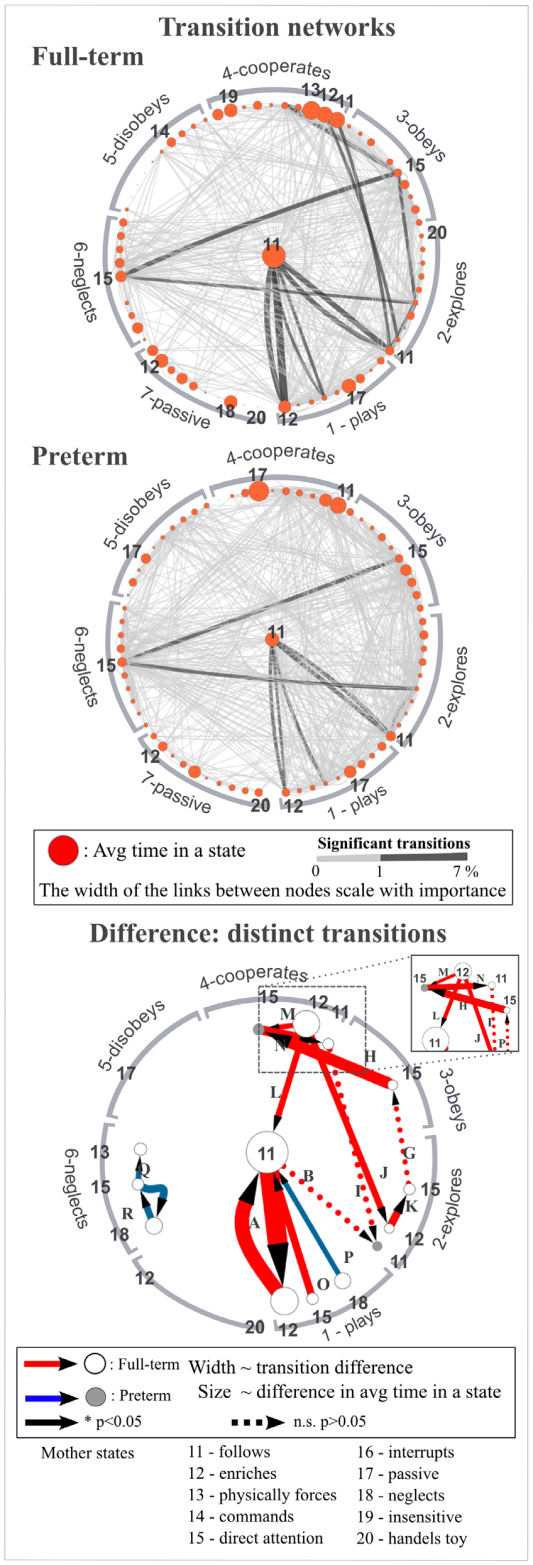
Interaction networks of the combined mother-infant behavioral transitions in full-term (top panel) and preterm (middle panel). Nodes (red circles) represent combined mother-infant behavioral states and their size is a measure of the average time spent in the combined state. The combined states are attributed an infant state (1 to 7, indicated in the outer ring) and a mother state, (11 to 20, listed to the right, and shown sequentially for each infant state). Links between the nodes indicate observed transitions from one combined state to another. The width and color of links change according to the transition rates, normalized within the group of either full-term or preterm (see the color scale). Transitions with probability less than 1% are bold. Bottom panel: The difference between the full-term and preterm transition networks. Arrows indicate transitions which have at least 0.5 transition rate in the full-term (red) or in the preterm (blue) network. Non-significant transitions are indicated by dotted arrows. The width of the links scale with the values of the transition differences and the node sizes scale with the absolute difference in time spent in the states. Dark grey nodes indicate states in which preterm infants/mothers spent the longer time whereas white nodes indicate states where full-term infants/mothers spent the longer time.

Behavioral transitions were visualized as a network using the online available network visualization software Cytoscape [33]. Each node in the network represents a combination of infant and maternal behaviors (termed “state”) and the links between the nodes represent transitions between these states (Figure 2). Most of the nodes are connected by links in both directions but for simplicity the arrows indicating the directions of transitions are not shown on the network figures. The most frequent state (infant plays/mother follows, 1–11) is highly connected in both the preterm and full-term groups; therefore we placed it in the center of networks in Figure 2. The networks do not contain any time components, it does not preserve the information how transitions (links between nodes) occur in time relative to each other (time-sequence). Each transition has been quantified by counting the number of occurrences of the particular transition in a given group and normalizing it by the total number of transitions observed in that group. The obtained value, termed “transition rate”, can be considered a percentage as it is the weight of a certain transition in relation to the total number of transitions. In this way, the transition rates are normalized to both the number of infants in each group, and the different length of the individual recordings. There are 62 links (1729 transitions) in the full-term behavioral network and 69 links (1864 transitions) in the preterm network. We found transition rates to be in the range of 0 to 5% for all transitions. The ‘other’ states (8 and 10) were omitted from the analysis because they cannot be linked to a specific behavior.

### Comparing Full-term and Preterm Behavioral State-transitions

In order to compare the interaction patterns of the two groups, their transition networks (described above) were subtracted from each other. In this way the distinctive transitions become visible. The subtracted interaction network has been generated by subtracting the transition rate of a given preterm transition from that of the same transition in the full-term network, and thus obtaining the difference between the two groups. Positive differences above 0.5 and negative differences below −0.5 are visualized in the subtracted network. This threshold has been set to be well above the majority of the links and is justified by the fact that most but not all the differences are statistically significant. Group-distinctive transitions are termed “distinct transitions”. In addition, the average time spent in each state was subtracted between the two groups and used for the scaling of node sizes. The differences have not been normalized.

### Statistical Significance of the Differences in the Transitions

The significance of distinct transitions between the groups were tested using χ^2^ tests, as the test of random networks can be used only for the transition with the highest occurrence. The transition with the highest occurrence (1–11→1–12, infant plays, mother follows/enriches) was tested against randomized networks to get a measure of the significance of this transition. The randomized networks were generated from the original interaction networks of the data by swapping the end-nodes of two randomly picked links while keeping the weight with the link. To generate one random network, 20000 link-swaps were performed, although a swap was only accepted if the resulting transitions were not present before the swap. In this way the general parameters for the network as the number of nodes, the number of links, and the number of connections each node has to other nodes in the network (degree distribution) are conserved [34]. 5000 random full-term and 5000 random preterm networks were derived. Single full-term and preterm randomized networks were subtracted in the same way as for the original data analysis. To see if the transition between the two combined states of 1–11 to 1–12 was a result of the network structure and not a finding in the data we recorded the number of times this transition favored full-term by at least the same amount as in the real data. The *p* value is then this number divided by the total number of tests. The observed strength of the full-term transition from 1–11 to 1–12 is likely not accidental (p<0.005).

### Sequence Analyses of Behaviors

Three previous transitions were analyzed starting from a “*distinct transition*” in order to detect the sequences of group-distinctive behaviors. All the states leading up to a distinct transition were recorded, and the occurrence of each state was counted. For each of these states, we again recorded which state happens right before, how many times each state happens, and so on. From this we could see if there is a certain pattern in previous transitions of states, leading up to the transition of interest. We could also generate a rate (in percentage of all states) of transitions leading up to the specific transition.

## Results

### Structural Features of Interaction Networks

**Figure 3:**
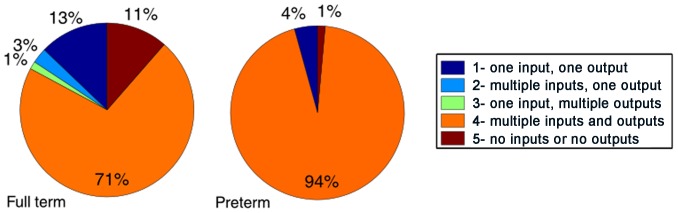
Connectedness of nodes in the full-term (left) and preterm (right) infant-mother interaction networks. A coarse-grained degree distribution analysis has been performed on the data, separating the combined mother-infant behavioral states into the 5 categories listed to the right. The 5 categories separate the states into the very sparsely connected states which have either one input and one output (1) or either no input or no output (5), states which have multiple inputs but only one output (2), states which have only one input but multiple outputs (3) and finally those which have multiple inputs and outputs (4).

The number of links connected to any given node (connectivity) in the network will fall into one of the following five categories: (1) one incoming and one outgoing link, (2) multiple incoming and one outgoing links, (3) one incoming and multiple outgoing links, (4) multiple incoming/outgoing links, and (5) no incoming/outgoing link (Figure 3).

The nodes were largely connected across the networks in both groups and very few sub-network structures were found. A sub-network would suggest that certain behavioral states and transitions would be isolated from the rest of the network and only reachable through the connecting node. The majority of nodes have multiple incoming and outgoing links in both the preterm and the full-term networks (Figure 3), however, the preterm network has significantly higher fraction of nodes with high connectivity than the full-term network, χ^2^ (1, N = 131) = 12.6, *p* = 0.00038. Higher connectivity suggests higher variability in behavioral transitions. The two networks show differences in the occurrence of the other four possible node-statuses (Figure 3), which occur more often in the full-term group. However, only the difference in the number of nodes which are not linked to any other nodes is statistically significant, χ^2^ (1, N = 131) = 5.51, *p* = 0.019. Most of these nodes correspond to states where the infant is passive (7–17, 7–19, 7–20) or disobeys (5–17, 5–18, 5–19), and to states where the mother is passive (3–17, 5–17, 7–17) or inappropriate (5–19, 7–19). Nodes corresponding to the “defies” behavior in infants are generally less connected in the full-term network (Figure 2, top and middle panels). The structural features of the interaction networks are summarized in Table 1.

**Table 1:** Structural features of the preterm and full-term interaction networks.

Full-term network	Preterm network
Nodes are largely connected across the network	
The majority of nodes have multiple incoming and outgoing links	
More nodes have no connection to other nodes	More nodes are connected to other nodes
Nodes corresponding to the ‘defies’ behavioral category in infants are less connected	More nodes have high connectivity

### Group-distinctive Transitions

The 6 most frequent transitions (A–F) were common in the two groups (see also Tables 2 and 3):

**Table 2:** The most frequent behavioral transitions in the preterm group identified by network analysis (Figure 2, middle panel).

Code	Mother	Infant	Direction	Mother	Infant
<A>	follows (11)	plays (1)	↔	enriches (12)	plays (1)
<B>	follows (11)	plays (1)	↔	follows (11)	explores (2)
C>	follows (11)	plays (1)	→	directs (15)	plays (1)
D>	follows (11)	explores (2)	→	directs (15)	explores (2)
E>	directs (15)	explores (2)	→	directs (15)	neglects (6)
F>	directs (15)	neglects (6)	→	directs (15)	obeys (3)

Directions of behavioral transitions are also indicated in the codes (< and >).

**Table 3:** The most frequent behavioral transitions in the full term group identified by network analysis (Figure 2, top panel).

Code	Mother	Infant	Direction	Mother	Infant
<A>	follows (11)	plays (1)	↔	enriches (12)	plays (1)
<B>	follows (11)	plays (1)	↔	follows (11)	explores (2)
<C>	follows (11)	plays (1)	↔	directs (15)	plays (1)
D>	follows (11)	explores (2)	→	directs (15)	explores (2)
E>	directs (15)	explores (2)	→	directs (15)	neglects (6)
F>	directs (15)	neglects (6)	→	directs (15)	obeys (3)
G>	directs (15)	explores (2)	→	directs (15)	obeys (3)
H>	directs (15)	obeys (3)	→	directs (15)	cooperates (4)
I>	follows (11)	cooperates (4)	→	follows (11)	explores (2)
J>	enriches (12)	cooperates (4)	→	enriches (12)	explores (2)

Directions of behavioral transitions are also indicated in the codes (< and >).

A While the mother and the infant are involved in a play initiated by the infant, the mother elaborates it occasionally.
B The infant stops being involved in a game and starts a new activity or the infant starts a new game and gets involved in it, while the mother follows his switch.
C The mother stops following the infant’s activity and attempts to direct the infant’s attention to a new toy, while the infant does not change behavior. Also vice versa in the full-term group: the mother stops directing the infant and starts to follow his/her activity.
D The infant starts a new activity, and the mother stops following him/her and tries to redirect his/her attention to the object of her own interest.
E The infant chooses a new toy, the mother attempts to direct his/her attention to another toy but the infant starts to neglect the mother.
F The infant neglects the directing mother but subsequently accepts the mother’s idea without sign of joy.

Subtraction of the two networks (Figure 2, bottom panel) revealed the most prominent differences in the occurrences of transitions. Group distinctive behavioral transitions (Full-term: G–N; Preterm: O–Q) are described below:

G The mother directs the infant, who is initiating a new activity. The infant stops initiating and obeys the mother’s direction (accepts her idea without sign of joy).
H The mother initiates and directs the activity, and the infant plays according to the mother’s idea joylessly. Subsequently the infant shows a signs of enjoying the activity.
I The mother initiates an activity and the infant plays according to the mother’s idea happily, while the mother follows him. Subsequently the infant initiates a new game and the mother follows his switch.
J The infant plays according to the mother’s idea and shows signs of joy while the mother enriches the activity. Then the infant chooses a new toy, and the mother enriches his/her activity.
K The infant chooses a new toy, and the mother enriches his/her activity. Subsequently the mother directs the infant’s attention to another object.
L The mother enriches the infant’s activity, and the infant plays happily the game suggested by the mother. Then the infant changes his activity according to his idea, and the mother follows him/her.
M The mother enriches infant’s activity, and the infant plays happily the game suggested by mother. Then the mother directs the infant’s attention to another object.
N The mother directs the infant’s attention to a new activity, and the infant happily plays along. Then the mother stops directing and follows the infant, who keeps playing.

The statistical significance of these differences was evaluated using χ^2^ tests (Tables 4 and 5). Based on the results of χ^2^ tests, transitions A, C, H, J, K, L,M, and N occur significantly more often in the full-term group than in the preterm group, and G and I show a tendency for that. In transitions A, B, C, I, J, L, and N the mother adapts or switches to adapt to the infant’s activity, while in transitions G and H the infant accepts the mother’s idea.

**Table 4:** Behavioral transitions which have at least 0.5 higher transition rates in the full term group.

Code	Mother	Infant	Direction	Mother	Infant	χ^2^	*p*
<A>	follows (11)	plays (1)	↔	enriches (12)	plays (1)	→16.43←11.39	0.000050.0007
B>	follows (11)	plays (1)	→	follows (11)	explores (2)	1.33	0.2488
<C	follows (11)	plays (1)	←	directs (15)	plays (1)	7.34	0.0067
G>	directs (15)	explores (2)	→	directs (15)	obeys (3)	3.07	0.0797
H>	directs (15)	obeys (3)	→	directs (15)	cooperates (4)	9.65	0.0019
I>	follows (11)	cooperates (4)	→	follows (11)	explores (2)	3.78	0.0518
J>	enriches (12)	cooperates (4)	→	enriches (12)	explores (2)	4.59	0.032
K>	enriches (12)	explores (2)	→	directs (15)	explores (2)	13.40	0.00025
L>	enriches (12)	cooperates (4)	→	follows (11)	plays (1)	7.27	0.007
M>	enriches (12)	cooperates (4)	→	directs (15)	cooperates (4)	6.96	0.0083
N>	directs (15)	cooperates (4)	→	follows (11)	cooperates (4)	6.49	0.0108

Results of χ^2^ tests and the corresponding right-tail probability values (1, N = 3593) are shown for each transition. Directions of behavioral transitions are also indicated in the codes (< and >).

**Table 5:** Behavioral transitions which have at least 0.5 higher transition rates in the preterm group.

Code	Mother	Infant	Direction	Mother	Infant	χ^2^	*p*
O>	directs (15)	neglects (6)	→	forces (13)	neglects (6)	5.00	0.025
P>	neglects (18)	plays (1)	→	follows (11)	plays (1)	6.45	0.011
<Q>	directs (15)	neglects (6)	↔	neglects (18)	neglects (6)	→9.69←9.45	0.00180.0021

Results of χ^2^ tests and the corresponding right-tail probability values (1, N = 3593) are shown for each transition. Directions of behavioral transitions are also indicated in the codes (< and >).

Based on the results of χ^2^ tests, transitions O, P, and Q can be considered to occur significantly more often in the preterm group than in the full-term group. None of these distinctive preterm transitions belong to the high frequency transitions:

O The infant neglects the directing mother, and subsequently the mother applies physical force. 30% of the preterm dyads have this transition (9 out of 30) and 4 out of the 9 dyads have this transition multiple times, compared to the full-term group where it occurs in only 4 of the 42 dyads, one of them having it twice.
P The infant plays based on his/her own idea while the mother does not pay attention to him/her and is actively engaged in another activity. Then the mother gets involved in the infant’s play. This transition occurs only twice in the full-term group (in 2 of the 42 dyads), and 12 times in the preterm group (in 6 of the 30 preterm dyads, and 3 of these have it more than once).
Q The participants neglect each other; there is no relationship between them. Subsequently the mother directs infant’s attention to a toy. Vice versa: the mother directs the infant, and the infant neglects the mother, and subsequently the mother disengages and starts to do something else, resulting in no relationship between the two. It happens very rarely (only 4 times) in the full-term group (6–18→6–15 in the case of 1 infant, and 6–15→6–18 for 3 infants). However, we found at least one transition in 30% of mother-preterm infant observations, and in 23% of the cases we observed more than one transition.

To get an insight how the distinctive preterm transitions affect the mother-infant interaction, we asked how often and how fast harmonious play (1–11 or 1–12) was developed after the O and Q transitions (P is a transition to 1–11).

When harmonious play is reached after transition O (6–15→6–13) it occurs in about 80 s (mean: 79.7 s, SD: 53.6 s) in the preterm group, and in about 21 s in the full-term group (mean: 20.67 s, SD: 2.52 s). 18.75% of the observed preterm O transitions (3 out of 16) and 40% of the O transitions in the full-term group (2 out of 5) was not followed by harmonious play within the recorded time.

In the full-term group a harmonious state (1–11 in all the cases) was reached relatively soon after the transition Q (mean = 29 s, SD = 25 s). In the preterm group transition <Q> occurred more frequently (*p*<0.05). We found at least one <Q> transition in 30% of the mother-preterm infant observations, and in 23% of the cases we observed it more than once. In these transitions infants neglect their mother’s attention directing attempt, for which mothers of preterms often respond by withdrawing themselves from the interaction (neglecting the infant), and then trying to direct the infant’s attention again. Interestingly, in the preterm group the 6–18→6–15 (mother switches from neglecting to directing the infant while infant neglects mother) transition led to harmonious play (1–11 or 1–12) after only ∼2 minutes (117 s) or longer (mean = 248 s, SD = 91 s), and in 21% of the cases the interaction never returned to harmonious after this transition. In the case of the 6–15→6–18 (Q>) transition (mother switches from directing to neglecting the infant while infant neglects mother), we found only one occasion where harmonious play (1–11) was reached in a short time (10 seconds), which represents about 5% of all the cases. Our data shows that in the preterm group the 6–15→6–18 (Q>) transition is one of the most unsuccessful maternal transitions from the 6–15 state. Results are summarized in Table 6.

**Table 6:** Characteristics of behavioral transitions.

Full-term group	Preterm group
The most frequent transitions are identical	
Transitions A, C,H,J,K,L, and M occur more frequently	Transitions O,P, and Q occur more frequently
	It is less probable and takes longer time to reach harmonious play after transitions O and Q.

### Comparing Full-term and Preterm Behavioral State Transition Sequences

**Figure 4:**
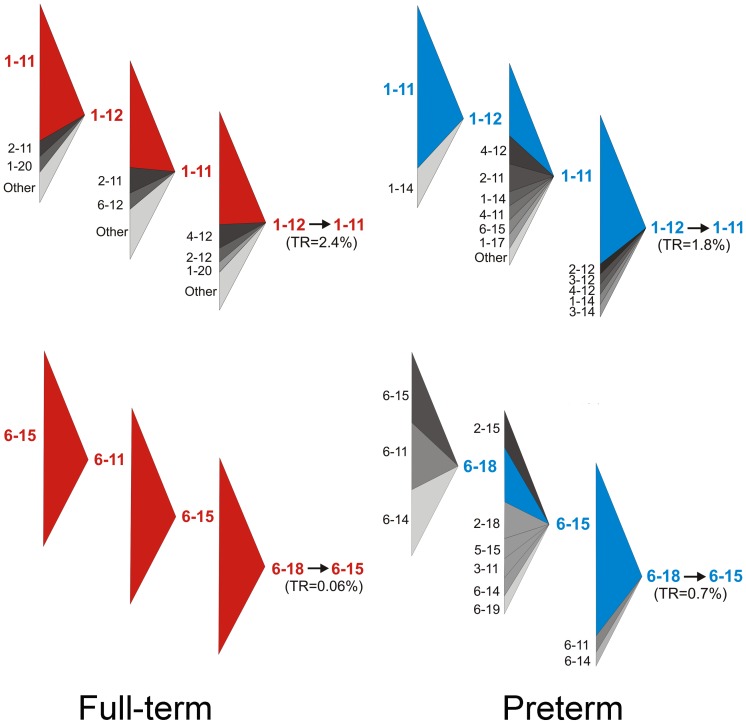
**Transition paths in the full-term (left panel) and preterm (right panel) groups preceding the transitions 1–12→1–11 and 6–18→6–15.** Transition rates (TR) are indicated. The large triangles show the distribution of states preceding the above transitions, where the baselines of the large triangles are divided proportionally to the occurrences of the states. The most frequent state (if there is one) is colored red or blue, other states are colored by different shades of grey. Only states occurring with ≥5% frequency are shown. The 6–18→6–15 transition sequence in the full-term group represents a single event.

The subtracted transition network suggests that there are potential distinctive paths in the system although the network on its own does not reveal the time sequence of transitions. In Figure 4 we show the distribution of states preceding a selected distinctive full-term (<A, 1–12→1–11) and distinctive preterm transition (<Q, 6–18→6–15). Transition <A>, which is the most frequent transition in this study, occurs in the full-term group more often than in the preterm group, and is often periodic. In this transition the infant plays based on his/her own idea, while the mother alternates between following and enriching his/her activity. Interestingly, during the sequence preceding the 1–12→1–11 (<A) transition mothers of full-term infants are predominantly in states 11 (follows), 12 (enriches), or 20 (handles toy), while mothers of preterm infants often can be found in state 14 (commands), 15 (directs attention) by both controlling the infant’s activity or 17 (being passive). Similar to the full-term group, in the preterm group the most likely preceding transition of 1–12→1–11 (<A) is 1–11→1–12 (A>) and vice versa (Figure 4).

Transition <Q>, which was found only once in the full-term group, is likely to happen periodically in the preterm group (mother directs/neglects infant while infant neglects mother, Figure 4). It was preceded by states where the infant was in the ‘neglect’ state in all cases, and most of the cases the mother was trying to direct the attention of the neglecting infant (6–15). This non-beneficial pattern of interaction between the mother and the infant is difficult to break once the mother and infant have entered it.

## Discussion

In this work we present a novel approach for analyzing mother-infant interactions, focusing on behavioral changes. The method was applied to compare the interaction of mothers with 12 months old preterm and full-term infants. The most frequent behavioral transitions were the same in both groups (A to F, Tables 2 and 3). The states with the infant playing and the mother following or enriching his/her activity occurred remarkably often. This interaction is often considered to be optimal in the western cultures [35]. In such cases the infant has the choice of what to play, and the mother stays involved in the interaction and helps to maintain the infant’s attention by occasionally enriching and elaborating his/her ideas. This maternal behavior is favorable in various aspects: it (i) enhances the infant’s focused attention by keeping him/her longer in a certain activity, (ii) enriches the infant’s knowledge and repertoire of skills, (iii) allows the infant to experience that he is an able-to-act individual, and (iv) provides mutual joy and satisfaction in the interaction.

Mostly mothers adjusted to the infant’s activity. This finding is in agreement with the observation of van Beek [16], who called this phenomenon ‘infant dominance’. However, occasionally infants followed mothers. Overall, both partners’ contribution is needed to create a harmonious interaction by accepting each other’s temporary leading role.

### Group Differences in Behavioral Transitions

Beside the major similarities, preterm dyads showed differences in the mother - infant interactions one year postpartum. We analyzed the possible patterns of transitions, i.e. how many different behaviors do precede and follow a given behavior. Interaction patterns are generally diverse in both groups, the majority of behavioral states can be reached from several different states, and can also lead to many different behaviors. However, significantly more behavioral states belong to this category in the preterm group (94% vs 71%, Figure 3). Behavioral states in the preterm group have generally higher connectivity, i.e. transition-paths are more diversified, suggesting that interaction sequences in the preterm group are more heterogeneous compared to the full-term group. Also, there are several behavioral states which occur only in the preterm group (Figure 3). In these states the infant is either passive or defies, and the mother is passive or inappropriate. Our results generally suggest that interaction of full-term infants and their mothers are more focused and harmonious, while the preterm transition pattern is more evenly spread. The method was also able to capture differences in the occurrences of certain behavioral transitions between the two groups. We identified 8 significant distinctive transitions which occurred substantially more frequently in the full-term group, and 3 distinctive transitions for the preterm group. Our results suggest that full-term infants spend more time playing based on their own ideas than their preterm peers, and transitions occur more frequently between playing, cooperating, exploring and obeying (Table 4). The major difference in the maternal behaviors is that the transition pattern of mothers of full-term infants is focused on three states: following and enriching the infant’s activity and directing the infant’s attention to her ideas. These transitions are more frequent than in the case of mothers of preterm infants. Our results on a low-to-medium risk preterm sample support the conclusions of several previous reports which found that the interactions of preterm dyads are less harmonious [10], [13], [14], [36], [37]. In the more disharmonious transitions (O, Q) mothers attempted to direct infants’ attention, when infants are very obviously not open for a new activity.

### Maternal Intrusiveness and Disengagement in the Preterm Group

Our findings do not support previous findings that mothers of preterm infants’ would be either more [14], [15] or less [10], [13] active, than mothers of full-term infants. Using network analyses on micro-analytic data rather suggests that during the interaction occasionally they can become both active (intrusive) and neglecting (disengaged).

Several studies observed elevated maternal intrusiveness among mothers of preterm infants [38], but according to our best knowledge, none of them examined closely how a 1-year old infant handles maternal intrusiveness. Infants of our preterm sample often do not pay attention to their attention directing mother, instead they neglect them. This behavior is similar, but more conscious, to that of young infants, who show gaze aversion to maternal overstimulation or attention attracting activity [18], [39]. The neglecting behavior of the infant can be an attempt to cope with the emotional distress caused by the intrusive mother. As a response to the neglecting behavior of their infants, mothers often increased control over the infant by using physical force (O) or disengaged from them (Q) (e.g. clean up the room). After these transitions harmonious play rarely developed, and even if it did, after a prolonged period, presumably because both infants and mothers got frustrated.

Much of the mother-infant interaction research has been aimed at better understanding maternal intrusiveness, and not much effort has been focused on examining the effects of maternal disengagement. Neglecting is not equivalent with the dimension of being non-responsive or active/passive, which got relatively high attention in the past decades [14], [15]. According to our coding-definition, a neglecting mother, despite of the instruction of the researcher, does not play with the infant, and actively involved in doing something else (e.g. tries to contact the cameraman, or cleans up the room).

Previous studies suggested that unpredictable alternation of maternal behavior between intrusiveness and disengagement may be particularly detrimental to the development of a young child, because they cannot anticipate and engage accordingly [40]. Despite its infrequency, negative control and maternal intrusiveness and hostility in the early mother–infant interaction can most likely be associated with behavioral and emotional symptoms of the child. The directive maternal behavior was also found to be associated with poorer language development [41]–[43].

### Conclusion

Our approach allowed an in-depth insight into the mother-infant interaction unattainable using the traditional methods of psychology. In addition to corroborating the existing view of the importance of preterm birth in mother-infant interactions, our findings supplemented the picture with additional details. In the context of mother and infant playing together, the most frequent behavioral transitions did not differ in the two groups: infant playing or exploring with mother following, enriching or directing. However, the transitions in the preterm dyads were found to be more diverse compared to their full-term counterparts, and they were also unfavorable as they tended to make the interactions disharmonious (mother neglecting, directing or forcing the infant). Because these maladaptive maternal behavioral changes are likely to place the infant at risk for later emotional, cognitive and behavioral disturbance, future cross-cultural research with larger samples is needed to confirm our conclusions. Also, longitudinal studies should clarify how the coupling of an over-sensitive infant with an intrusive/disengaged mother affects the development outcome.
